# Comparison of Computerized Cardiotocography Parameters between Male and Female Fetuses

**DOI:** 10.3390/medsci7030050

**Published:** 2019-03-21

**Authors:** Elena R. Magro-Malosso, Giovanni Sisti, Viola Seravalli, Tomi T. Kanninen, Martina Aldinucci, Mariarosaria Di Tommaso

**Affiliations:** 1 Department of Health Science, Division of Pediatrics, Obstetrics and Gynecology Careggi Hospital, University of Florence, 50121 Florence, Italy; elenaritamag@gmail.com (E.R.M.-M.); violaseravalli@gmail.com (V.S.); martina.aldinucci@gmail.com (M.A.); 2 Department of Obstetrics and Gynecology, Lincoln Medical and Mental Health Center, Bronx, NY 10451, USA; gsisti83@gmail.com; 3 Department of Obstetrics and Gynecology, Richmond University Medical Center, Staten Island, NY 10310, USA; ttkanninen@gmail.com

**Keywords:** fetal sex, cardiotocography, fetal heart rate, computerized analysis, uncomplicated pregnancy

## Abstract

Fetal sex has been identified as an important factor influencing pregnancy outcomes, but its impact on fetal heart rate (FHR) variability in uncomplicated pregnancies is still unclear. The objective of the study was to assess short-term variability (STV) and other computerized cardiotocography (cCTG) parameters in relation to fetal sex during fetal antepartum surveillance. We retrospective compared cCTG parameters of male and female fetuses in uncomplicated singleton pregnancies at term. In addition to univariate analysis, a multivariate analysis was performed taking into account maternal characteristics. A total of 689 cCTG recordings were analyzed: 335 from male fetuses and 354 from female fetuses. Analysis of cCTG results by fetal sex showed no significant difference in percentage of signal loss, number of contractions, movements, accelerations and decelerations, long-term variability (LTV), and STV at both uni-and multivariate analysis. There was a statistically significant difference for baseline FHR at the univariate analysis, which was not confirmed by a multivariate analysis. Our results suggest that fetal sex did not affect cCTG parameters in uncomplicated term singleton pregnancies, and therefore it does not need to be taken into account when interpreting cCTG in physiological conditions.

## 1. Introduction

Fetal sex has been identified as an important factor influencing both fetal and maternal outcome. The hormonal secretion of various substances during pregnancy is influenced by fetal sex [1,2,3]. Specifically, some placental functions are sex-specific with different pathways of gene expression, proteins, and steroids according to the sex of the fetus. This sex-specific difference in the feto-placental immune system results in the adoption of different strategies by males and females to face the same adverse maternal environment. In general, males have a greater risk of adverse outcome because of minimal placental adjustment with the aim of ensuring continued growth [4]. Many studies support this statement, showing an increased risk of preterm birth, cesarean delivery, failed induction of labor, perinatal mortality, and gestational diabetes in pregnant women with male fetuses [4,5]. Conversely, the multiple placental adaptations in gene and protein expression in female fetuses result in increased survival, at the expense of reduced fetal growth and resulting in an increased risk of developing gestational hypertensive disorders [4]. Thus, placental function seems to be sensitive to fetal sex, and the feto–maternal interaction therefore seems to be reflected in a placental response that differs between males and females. Given the difference in pregnancy outcome between male and female fetuses, various authors have examined fetal heart rate (FHR) variability and other parameters of fetal cardiotocography (CTG) in order to identify differences between sexes. However, most of these studies assessed FHR differences during labor or in particular conditions related to individual fetal behavioral states [6,7,8,9]. Therefore, findings of these studies cannot be generalized to the whole antepartum period. Moreover, previous studies often examined indices of the FHR that are difficult to reproduce, such as power spectral density (PSD) and approximate entropy (ApEn) [6,7,10,11].

The objective of this study was to assess short-term variability (STV) and other additional computerized CTG (cCTG) parameters in relation to fetal sex during fetal antepartum surveillance in a large population of uncomplicated singleton pregnancies at term.

## 2. Materials and Methods

We conducted a retrospective analysis of computerized CTG tracings in women with uncomplicated singleton pregnancies between 37 and 40 weeks of gestation, who were referred to the outpatient clinic of the Careggi University Hospital in Florence, Italy, between January and December 2014. The last tracing of male and female fetuses during the antepartum period, before labor, was selected. The regional public health care offers fetal antepartum surveillance in the third trimester to all pregnant women. In our hospital, it is common to do a cCTG even in low-risk pregnancies after 37 weeks of gestation. This study was approved by the Local Ethics Committee. We adopted the definition of Chappell and colleagues for uncomplicated pregnancy: “a normotensive pregnancy, delivered at >37 weeks, resulting in a live born baby who was not small for gestational age, and did not have any other significant pregnancy complications” [12]. We have complied with the World Medical Association Declaration of Helsinki regarding ethical conduct of research involving human subjects. Results of cCTG recordings were obtained from the Sonicaind FM 8000 and matched with the electronic patient records (Argos 3.34 Dedalus SpA, Florence, Italy). External CTG was used for monitoring the FHR for 20 min and the activity of the uterine muscle by two transducers placed on the maternal abdomen: one above the fetal heart level and the other at the uterine fundus. When evaluating cCTG data obtained from the Sonicaid FM800E (Huntleigh Healthcare Ltd, Cardiff, UK), the following information was evaluated: baseline FHR (expressed in beats per minute), number of uterine contractions, number of accelerations and/or decelerations, duration of episodes of long-term variability (LTV) in minutes, STV in milliseconds, percentage of signal loss, and number of fetal movements. The FHR parameters considered in the study was defined according to FHR monitoring guidelines, Sonicaid computer systems [13]. Only one tracing per fetus was included (the last tracing before the onset of labor). Data on mode of delivery and neonatal outcome were also collected and compared between male and female sex. Categorical variables were compared using the chi-squared test, and continuous variables were compared using the Mann–Whitney test, given the non-parametric distribution of the data. For multivariate analysis we used a multiple linear regression model with a binary outcome (dependent variable) and several predictors (independent variables). Specifically, the multivariate analysis pooled together the cCTG results to correct for possible confounders during data interpretation. The independent variables were the following: baseline FHR, number of uterine contractions, number of accelerations, number of decelerations, LTV (min), STV (ms), signal loss (%), and number of fetal movements. The binary outcome was represented by fetal sex (male vs female). The software used for the statistical analysis was SPSS 23.0 (IBM, Chicago, IL, USA). The last antepartum computerized CTG tracing of male and female fetuses in uncomplicated singleton pregnancies was included. Exclusion criteria were: gestational age <37 weeks or after 41 complete weeks, congenital malformations, maternal comorbidities, or pregnancies complicated by obstetrical pathological conditions such as hypertensive disorders, fetal growth restriction, and gestational diabetes.

## 3. Results

A total of 689 cCTG recordings, obtained from 689 pregnancies, were analyzed, with 335 from male fetuses and 354 from female fetuses. Maternal characteristics are presented in Table 1. Median gestational age at cCTG recording was the same between male and females fetuses. Four cases of males had a missing description of maternal obstetric history, 3 males had missing ethnicity value; 4 cases of females had missing obstetric history and maternal ethnicity. A statistically significant difference was found in the ethnicity of the patients at the univariate analysis, which was not confirmed by multivariate linear regression (data not shown). Analysis of cCTG results by fetal sex showed no significant difference in percentage of signal loss, number of contractions, movements, accelerations and decelerations, LTV, and STV at both uni- and multivariate analysis. There was a statistically significant difference for the baseline FHR at the univariate analysis (Table 2, *p* < 0.05), which was not confirmed by a multivariate analysis. Delivery mode and neonatal outcome of males and the females are summarized in Table 3. Mean birth weight was significant higher in males than in females (*p* < 0.05). There was no difference in the Apgar score, umbilical cord pH, base excess (BE), gestational age at delivery, and mode of delivery between sexes.

## 4. Discussion

Current available evidence shows that cardiotocography performed in specific conditions is affected by fetal sex. Bernardes et al. studied specific indices of linear and complex FHR variability in relation to fetal behavioral states, during the antepartum period of normal term pregnancies, and found significant sex differences [6]. The same authors found more marked changes in FHR in female fetuses, in relation to progression of labor and during the minutes preceding delivery, particularly in fetuses with academia [7]. Similar results were reported when computerized CTG was used for FHR analysis during the last hour before delivery in term fetuses [8]. Fetal heart rate changes that differ between male and female fetuses have also been observed after maternal chocolate intake [14]. Additionally, gestational age should be considered when defining reference ranges for FHR indices in systems of computerized analysis. In a large retrospective study using antepartum FHR recordings of male and female fetuses, with normal pregnancy outcome, mean FHR decreased significantly throughout gestation, whereas most variability indices increased. Gestational ages of this study ranged between 25 and 40 weeks [11]. In our study we considered only uncomplicated singleton pregnancies between 37 and 40 weeks of gestation, reducing significantly the possible influence of gestational age on FHR variability in both sexes. A retrospective cross-sectional study using antepartum tracing of singleton fetuses with normal pregnancy outcomes provided reference values for CTG parameters throughout pregnancy from 24 to 40 weeks of gestation. FHR parameters were evaluated throughout pregnancy by comparing four different gestational age intervals: 24–27 weeks, 28–31 weeks, 32–35 weeks, and 36–40 weeks. Comparisons were made separately in female and male fetuses for variables showing statistical significant differences between these two groups [15]. We compared FHR parameters between male and female fetuses in a range of 37–40 weeks reporting no statistical difference.

We examined cCTG parameters of male and female fetuses in singleton pregnancies during the antepartum period, before labor, without focusing on a particular maternal or fetal condition, in order to clarify the influence of fetal sex on CTG results. Only uncomplicated term pregnancies have been included to avoid confounders such as pathological conditions or preterm gestational age.

No significant difference was found in most of the computerized CTG parameters, such as number of accelerations and/or decelerations, duration of episodes of LTV, STV, and number of fetal movements, at either uni-and multivariate analysis. Although there was a statistically significant difference in the baseline FHR between male and female fetuses at univariate analysis, this was not confirmed at multivariate analysis. Our results are consistent with a previous prospective study in which the authors found no sex difference in FHR variation using cCTG analysis. However, in that study the number of fetuses observed was extremely limited (44 males and 35 females) and the gestational age at monitoring was lower than the one we used (median 35 weeks) [16].

Cardiotocography is widely used to assess fetal well-being and the comparison of cCTG versus traditional CTG showed a significant reduction in perinatal mortality with the computerized analysis (RR 0.20, 95% CI 0.04 to 0.88) [17]. The STV is one of the most used indicator of fetal metabolic status, as it correlates with a condition of fetal acidemia when significantly reduced [18].

Our results suggest that no sex-specific variations of fetal heart parameters are present in low risk pregnancies at term, when monitored outside the intrapartum period. Probably, the influence of fetal sex is mostly observed in complicated pregnancies, where sex has been observed to influence placental response to pathological conditions such as gestational hypertensive disorders, fetal growth restriction, and gestational diabetes [4]. Data on mode of delivery and neonatal outcome were similar in both male and female groups in our study, except for a higher birth weight in male fetuses.

The strength of our study is the accurate selection of the cases, since we excluded gestational disorders, low gestational age, maternal and fetal diseases, and peculiar times of pregnancy such as labor, which could have affected the CTG variables regardless of the sex of the fetus. However, it is possible that the development of some pathological conditions during pregnancy is associated with detection of differences in cCTG parameters between males and females which may not have been captured by our study design. Another limitation is that sex differences probably were not found because of the FHR parameters considered or for the type of computer system used for CTG analysis. Additionally, different definitions of the main FHR parameters are present according to the current FHR monitoring guidelines [13,19,20,21].

However, a computer system that follows the standard definitions of CTG features was used, providing an objective, quantitative, and consistent assessment of the CTG reading.

In conclusion, our results suggest that fetal sex does not affect cCTG parameters in uncomplicated term singleton pregnancies, and therefore it does not need to be taken into account when interpreting cCTG in physiological conditions. Conversely, future studies should investigate the impact of sex on cCTG parameters in pathological pregnancies, especially at earlier gestational ages.

## Figures and Tables

**Table 1:** Maternal characteristics according to fetal sex.

	Male Fetuses (*n* = 335)	Female Fetuses (*n* = 354)	*p*-Value
Gestational age at cCTG (weeks)	40 (38–40)	40 (38–40)	0.89
Maternal age (years)	34 (29–37)	33 (30–37)	0.57
Pre-pregnancy BMI (kg/m^2^)	22.1 (19.9–24.7)	22 (20–24.4)	0.91
Maternal obstetric history			
Nulliparous	199 (60.1%)	204 (58.3%)	
Parous with one previous birth	111 (33.5%)	116 (33.1%)	0.54
Parous with ≥2 previous births	21 (6.3%)	30 (8.6%)	
Maternal ethnicity—*n* (%)			
Caucasian	287 (86.4%)	280 (80%)	0.03
Latin American	9 (2.7%)	23 (6.6%)	
African	19 (5.7%)	18 (5.1%)	
Asian	17 (5.1%)	29 (8.3%)	

cCTG, computerized cardiotocography; BMI, body mass index. Continuous variables are expressed with median, 25th–75th percentile; categorical variables are expressed with *n*, percentage.

**Table 2:** Computerized CTG results according to fetal sex.

	Male Fetuses (*n* = 335)	Female Fetuses (*n* = 354)	*p*-Value (Univariate Analysis)	*p*-Value (Multivariate Analysis) *
Baseline FHR (bpm)	135 (128–141)	136 (130–142)	0.03	0.25
Uterine contractions (*n*.)	1 (0–2)	1 (0–2)	0.82	0.62
Accelerations (*n*.)	3 (2–5)	3 (2–5)	0.13	0.35
Decelerations (*n*.)	0 (0–0)	0 (0–0)	0.21	0.29
LTV (minutes)	8 (6–10)	8 (6–10)	0.18	0.82
STV (ms)	10.1 (8.1–12.5)	9.65 (8.1–11.7)	0.05	0.32
Signal loss (%)	1 (0–4)	1 (0–4)	0.35	0.76
Fetal movements (*n*.)	10 (4–24)	10.5 (2–28)	1	0.48

bpm, beats per minute; FHR, fetal heart rate; LTV, long-term variability; STV, short-term variability. Continuous variables are expressed with median, 25th–75th percentile. *multivariate analysis, pooling together maternal characteristics (Table 1) and CTG results.

**Table 3:** Neonatal outcome according to fetal sex.

	Male Fetuses	Female Fetuses	*p*-Value
Birth weight (g)	3500 (3200–3817)	3360 (3135–3700)	0.002 *
Apgar score at 1 min	9 (9–9)	9 (9–9)	0.074 *
Apgar score at 5 min	10 (9,10)	10 (9,10)	0.687 *
Umbilical cord pH	7.3 (7.2–7.3)	7.3 (7.2–7.3)	0.388 *
Base excess	−4.3 (−7.3–−2.0)	−3.9 (−6.5–−1.6)	0.082 *
Mode of delivery—*n* (%)			
Vaginal delivery	248 (74%)	280 (79.1%)	0.116 **
Cesarean delivery	87 (26%)	74 (20.9%)	

Continuous variables are expressed with median, 25–75 percentile. * Mann–Whitney test. ** chi-squared test.

## Abbreviations

FHR	fetal heart rate
STV	short-term variability
cCTG	computerized cardiotocography
LTV	long-term variability

## References

[1] Bremme K., Eneroth P. (1983). Fetal sex dependent hormone levels in early pregnant women with elevated maternal serum alpha-fetoprotein. Int. J. Gynaecol. Obstet..

[2] Houghton D.J., Shackleton P., Obiekwe B.C., Chard T. (1984). Relationship of maternal and fetal levels of human placental lactogen to the weight and sex of the fetus. Placenta.

[3] Sowers S.G., Reish R.L., Burton B.K. (1983). Fetal sex-related differences in maternal serum alpha-fetoprotein during the second trimester of pregnancy. Am. J. Obstet. Gynecol..

[4] Clifton V.L. (2010). Review: Sex and the Human Placenta: Mediating Differential Strategies of Fetal Growth and Survival. Placenta.

[5] Di Renzo G.C., Rosati A., Donati Sarti R., Cruciani L., Cutuli A.M. (2007). Does Fetal Sex Affect Pregnancy Outcome?. Gend Med..

[6] Bernardes J., Gonçalves H., Ayres-de-Campos D., Rocha A.P. (2008). Linear and complex heart rate dynamics vary with sex in relation to fetal behavioural states. Early Human Dev..

[7] Bernardes J., Gonçalves H., Ayres-de-Campos D., Rocha A.P. (2009). Sex differences in linear and complex fetal heart rate dynamics of normal and acidemic fetuses in the minutes preceding delivery. J. Perinat. Med..

[8] Dawes N.W., Dawes G.S., Moulden M., Redman C.W. (1999). Fetal heart rate patterns in term labor vary with sex, gestational age, epidural analgesia, and fetal weight. Am. J. Obstet. Gynecol..

[9] Yohai D., Baumfeld Y., Zilberstein T., Yaniv Salem S., Elharar D., Idan I., Mastrolia S.A., Sheiner E. (2017). Does gender of the fetus have any relation with fetal heart monitoring during the first and second stage of labor?. J. Matern. Fetal. Neonatal. Med..

[10] Gonçalves H., Fernandes D., Pinto P., Ayres-de-Campos D., Bernardes J. (2017). Simultaneous monitoring of maternal and fetal heart rate variability during labor in relation with fetal gender. Dev. Psychobiol..

[11] Chappell L.C., Seed P.T., Myers J., Taylor R.S., Kenny L.C., Dekker G.A., Walker J.J., McCowan L.M.E., North R.A., Poston L. (2013). Exploration and confirmation of factors associated with uncomplicated pregnancy in nulliparous women: prospective cohort study. BMJ.

[12] Pardey J., Moulden M., Redman C.W. (2002). A computer system for the numerical analysis of nonstress tests. Am. J. Obstet. Gynecol..

[13] Tranquilli A.L., Lorenzi S., Buscicchio G., Di Tommaso M., Mazzanti L., Emanuelli M. (2014). Female fetuses are more reactive when mother eats chocolate. J. Matern. Fetal. Neonatal. Med..

[14] Gonçalves H., Amorim-Costa C., Ayres-de-Campos D., Bernardes J. (2017). Gender-specific evolution of fetal heart rate variability throughout gestation: A study of 8823 cases. Early Hum. Dev..

[15] Amorim-Costa C., Cruz J., Ayres-de-Campos D., Bernardes J. (2016). Gender-specific reference charts for cardiotocographic parameters throughout normal pregnancy: A retrospective cross-sectional study of 9701 fetuses. Eur. J. Obstet. Gynecol. Reprod. Biol..

[16] Ogueh O., Steer P. (1998). Gender does not affect fetal heart rate variation. Br. J. Obstet. Gynaecol..

[17] Grivell R.M., Alfirevic Z., Gyte G.M., Devanen D. (2015). Antenatal cardotocography for fetal assessment. Cochrane Database Syst. Rev..

[18] Dawes G.S., Moulden M., Redman C.W. (1992). Short-term fetal heart rate variation, decelerations, and umbilical flow velocity waveforms before labor. Obstet. Gynecol..

[19] Ayres-de-Campos D., Bernardes J. (2004). Comparison of fetal heart rate baseline estimation by SisPorto 2.01 and a consensus of clinicians. Eur. J. Obstet. Gynecol. Reprod. Biol..

[20] Intrapartum Care: Care of the Healthy Woman and Their Babies during Childbirth. http://nice.org.uk/guidance/cg190.

[21] Royal College of Obstetricians and Gynaecologists (2001). The Use of Electronic Fetal Monitoring.

